# Sex Differences in Depression Caused by Early Life Stress and Related Mechanisms

**DOI:** 10.3389/fnins.2022.797755

**Published:** 2022-05-18

**Authors:** Xianquan An, Wanxu Guo, Huiying Wu, Xiying Fu, Ming Li, Yizhi Zhang, Yanlin Li, Ranji Cui, Wei Yang, Zhuo Zhang, Guoqing Zhao

**Affiliations:** ^1^Department of Anesthesiology, China-Japan Union Hospital of Jilin University, Changchun, China; ^2^Department of Anesthesiology, Second Hospital of Jilin University, Changchun, China; ^3^Jilin Provincial Key Laboratory on Molecular and Chemical Genetic, Second Hospital of Jilin University, Changchun, China; ^4^Department of Orthopedics, China-Japan Union Hospital of Jilin University, Changchun, China

**Keywords:** 5-HT, sex hormone, HPA axis, epigenetics, depression

## Abstract

Depression is a common psychiatric disease caused by various factors, manifesting with continuous low spirits, with its precise mechanism being unclear. Early life stress (ELS) is receiving more attention as a possible cause of depression. Many studies focused on the mechanisms underlying how ELS leads to changes in sex hormones, neurotransmitters, hypothalamic pituitary adrenocortical (HPA) axis function, and epigenetics. The adverse effects of ELS on adulthood are mainly dependent on the time window when stress occurs, sex and the developmental stage when evaluating the impacts. Therefore, with regard to the exact sex differences of adult depression, we found that ELS could lead to sex-differentiated depression through multiple mechanisms, including 5-HT, sex hormone, HPA axis, and epigenetics.

## Introduction

Major depression disorder (MDD) affects 17% of the population worldwide as a significant cause of disability (Wang et al., 2020). Depression is characterized by high incidence, a low diagnosis rate, and complex reasons. Increasing attention has been paid to the correlation between early life stress (ELS) and depression in adolescence and adulthood (Toledo-Rodriguez and Sandi, 2011).

Early life stress is defined as a series of adverse, stressful events occurring during the neonate, childhood, and adolescence period (Pervanidou et al., 2020), including physical or sexual abuse, neglection, and loss. Recent studies have shown that individuals who experience ELS are more likely to suffer from depression than those who do not (LeMoult and Gotlib, 2019). Whether it is the depression among the Uganda (Satinsky et al., 2021) or Iraq youth (Al Shawi et al., 2019), or the PTSD and TRD of American veterans (Aronson et al., 2020; Yrondi et al., 2021), ELS is regarded as a risk factor in depression (Macedo et al., 2019). The different contents of ELS (physical or sexual abuse, neglection, loss, etc.) may be correlated with specific psychological disorders in adulthood (Carr et al., 2013). The main factors influencing adverse effects in later life include the developmental time window suffering ELS, sex (Baker and Shalhoub-Kevorkian, 1999), and the content of the stress (Baker and Shalhoub-Kevorkian, 1999; Slavich and Sacher, 2019). Depression during adulthood is associated with ELS; moreover, there are more females with depression than males with depression (Avenevoli et al., 2015). Therefore, it is crucial to explore the sex differences in depression caused by ELS and its mechanism to reduce depression and develop a more effective treatment strategy.

The incidence rate of depression differs among different sexes. Before the stage of adolescent transition, the incidence of depression between both sexes is similar and relatively low, with about 3% of the children qualifying the diagnostic criteria of MDD over the past year (Merikangas et al., 2010). However, the incidence rate of MDD increased by approximately five times after adolescence, which is impressive, together with the significant sex differences. Adolescent girls are at least two times more likely to suffer from depression than boys (Avenevoli et al., 2015). In addition to sociological studies, scholars have proposed several mechanisms to explain the rapid increase in MDD risk of girls relative to boys (Gold, 2015), including pressure generation and change of hypothalamic pituitary adrenocortical (HPA) axis to pressure (Hammen, 2006). However, a single mechanism is unable to thoroughly explain the precise causes of depression by ELS. We will review the current research progress from the aspects of 5-HT, sex hormone, HPA axis, and epigenetics.

## 5-HT

5-HT is an essential neurotransmitter in emotional regulation whose function is mainly to regulate functional activities in the central nervous system, such as pain and analgesia, mental mood, sleep, body temperature, sexual behavior, pituitary endocrine, cardiovascular regulation, and somatic movement (Borrow and Cameron, 2014). A few studies have shown that 5-HT in brain tissue, especially in the amygdala, decreases after stress in early life (de Lima et al., 2020).

Serotonin transporter (5-HTT) is one of the critical regulators of serotonin neurotransmission (Vai et al., 2020), with the highest density in the raphe nucleus. It also mediates tryptophan hydroxylase (TPH), the rate-limiting enzyme for 5-HT synthesis (MacGillivray et al., 2010). The gene of 5-HTT locates in 17p13 and consists of 14 exons and 1 promoter. The variable nucleotide repeat of 5-HTTLPR/SLC6A4 is located at 1.400 bp upstream of the transcription initiation site. It consists of two common alleles, a short (s) variant with 14 copies and a long (L) variant with 44 bp repeat elements with 16 copies (Aslund et al., 2009). The S allele of 5-HTTLPR, together with childhood adversity, is associated with smaller hippocampal volume and depressive episodes (Kim et al., 2019). The polymorphism in the promoter region of the serotonin transporter gene (5-HTTLPR) is one of the research hot spots, but few studies explore its sex differences in depression induced by ELS.

### Studies on Humans

There is sex dimorphism in the serotonin system. First of all, compared with women, men show higher rates of 5-HT synthesis (Nishizawa et al., 1997), a significantly lower 5-HT (1A) receptor, and higher 5-HTT-binding potentials in a wide array of cortical and subcortical brain regions (Jovanovic et al., 2008). Women show a positive correlation between a 5-HT (1A) receptor and 5-HTT-binding potentials for the region of the hippocampus, a favorable response to SSRI than to tricyclic antidepressant (Kornstein et al., 2000). Besides, if serotonergic genes interact with other sexual dimorphic biological pathways, sex differences will increase exponentially, especially in early childhood (Caspi et al., 2003). Secondly, the studies using a questionnaire survey and gene sequencing showed that there was a strong relationship between past abuse and adolescent depression, regardless of sex; the interaction effect of G × E in girls with the SS allele showed that homozygous individuals with short allele would have a higher risk of depression when interacting with abuse. Among boys, no trend was even found in this regard (Collier et al., 1996). Thirdly, tests of umbilical cord blood of newborns experiencing prenatal stress (PNS) and the control group suggested that SLC6A4 methylation was higher in females than in males and was not affected by maternal stress or 5-HTTlPR genotype (Dukal et al., 2015). Dukal selected pregnant women who experienced PNS and took umbilical cord blood for genotype analysis. The results showed that only the sex of newborns was related to SLC6A4 methylation, no correlation between a genotype and ELS (Dukal et al., 2015). Fourthly, the tryptophan hydroxylase 2 (TPH2) gene encodes related rate-limiting enzymes in the biosynthesis of 5-HT. After having examined 291 patients with MDD and 100 healthy controls, Shen found that three CpG loci can predict the response to antidepressant treatment in different sexes. Qualified Childhood Trauma Questionnaire (CTQ) scores were significantly associated with a low level of DNA hypomethylation at TPH2-8-237 in male CpG sites (Shen et al., 2020). Fifthly, it is reported that at least 5 of the 14 kinds of 5-HT receptor subtypes are active in depression, 5-HT 1A, 5-HT 1B, 5-HT 4, 5-HT 6, and 5-HT 7 (Yohn et al., 2017). Women with MDD had significantly lower levels of the 5-HT1A receptor protein in the prefrontal cortex (PFC), while men had no change (Szewczyk et al., 2009), while not all measured gene methylation results are sex specific. Vijayendran studied the methylation of two CpG residues (cg22584138 and cg05951817) related to the SLC6A4 gene promoter (Kaffman and Meaney, 2007). Genotype and sexual abuse affected the methylation of cg22584138, while the methylation of cg05016953 was only affected by the history of sex abuse. Researchers have not found other factors affecting the methylation of CpG dinucleotides up to date. Sex differences were not emphasized (Vijayendran et al., 2012). What is more, monoamine oxidase A (MAOA) gene variation is also associated with risk of depression. MAOA is an X-linked gene that regulates monoamine neurotransmission by degrading serotonin, norepinephrine, and dopamine. There are limited studies on the sex differences between MAOA gene variation and ELS inducing depression. Melas et al. (2013) investigated the risk of mental disorder in people with childhood adversity, and found that adult MAOA-L women with childhood adversity had a higher risk of depression and the depressed women had a lower level of overall MAOA methylation, and that MAOA-L may be related to NR3C1 hypermethylation. Other studies have shown that monoamine oxidase-A–linked polymorphic region allelic variation (MAOA-uVNTR) was associated with decreased transcriptional activity, increased depressive symptoms, and poor sleep quality (Brummett et al., 2007). Another study of hospitalized patients with depression showed that genetic variations in the MAOA gene may influence the course of major depression by disrupting cortical limbic connectivity. Depressed MAOA-H carriers showed the weakest amygdala – prefrontal coupling in the study subgroup (Dannlowski et al., 2009). In conclusion, the low rate of 5-HT synthesis, the low amount of 5-HT1A in the cortex, the higher sensitivity of SS allele to stress environment, and the higher DNA methylation level of TPH2-8-237 in women may altogether contribute to higher risk of developing depression.

### Studies on Animals

The animal models of ELS used in the studies include maternal-infant nesting (MS), limited nesting, and the bedding material paradigm, and the study results also varied according to the animal type, pattern-making method, and the brain area detected. González-Pardo et al. (2020) used the MS depression model of rats, and found that 5-HT in the PFC, 5-HT, and 5-hydroxyindoleacetic acid (5-HIAA) in the hippocampus was increased, and the 5-HIAA/5-HT ratio was decreased after MS in females; in males, 5-HT was decreased in the PFC and was significantly increased in the striatum after MS. Liu et al. (2021) applied C57BL/6N mice to administer limited nesting and a bedding material paradigm to implement ELS. In pharmacological behavior experiments, female mice showed deeper depression and more sensitivity to ELS than male mice. Immunohistochemistic (IHC) staining showed that female serotonin (SERT) in accumbens (NAcc) and basolateral amygdala (BLA) were decreased significantly, and application of vortioxetine could help to attenuate this change (Liu et al., 2021). Arborelius used the MS model for the study, separating the rat cubs for 180 min (long-term mother separation; LMS) or 15 min (short maternal separation; BMS), and they found that, in the LMS group, the levels of 5-HT and 5-HIAA in the dorsal raphe nucleus (DRN) were significantly increased in females, and the levels of 5-HIAA and homovanillic acid in the nucleus accumbens (NAc) were also higher than those of the animal facility reared (AFR) and BMS groups. In the cingulate cortex, both LMS and BMS reduced the level of norepinephrine (NA). In addition, BMS mainly affected monoaminergic levels in the amygdala (Arborelius and Eklund, 2007). Besides, 5-HTT± heterozygous mice were used to study the variation of serotonin transporter 5-HTT/SLC6A4. The results showed that, in the aspect of depression-like behavior, postnatal stress (PS) could increase the depression-like behavior in 5-HTT± mice, but not in the wild-type; the basic level in 5-HTT± was lower than that of wild-type along with a more significant level in male offspring. Concerning gene expression, significant changes occurred in MARK and the neurotrophic protein pathway for both the 5-HTT± group and the PS group, but changes in the cytokine and Wnt signal pathway occurred in the heterozygous + PS group instead of the others, in which sex differences were not identified (van den Hove et al., 2011). We could infer that the 5-HTT± genotype is more sensitive to external stimulation, which was also confirmed by Houwing et al. (2020), who used fluoxetine as external stimulation. In addition, on the aspect of the serotonin transporter-linked polymorphism (5-HTTLPR), Schwandt genotyped macaques with 5-HTTLPR and evaluated the effects of genotypes, ELS, and sex on behavioral response. Males showed a higher level of aggression and social/courage than females. Besides, if peers also raised males carrying the S allele in early adversity, their attack risk increased significantly (Schwandt et al., 2010). On the aspect of the 5-HT1A receptor, Spinelli et al. (2010) measured the density of 5-HT1A by positron emission tomography (PET) in young rhesus monkeys raised by females and peers, respectively. The examination showed that the density of 5-HT1A was decreased in peer-rearing rhesus, suggesting that the decrease of 5-HT1A receptor density during development may be a factor in increasing vulnerability (Spinelli et al., 2010). The chronic mild stress (CMS) depression model showed decreased 5-HT1AR mRNA expression in the lateral orbitofrontal cortex (OFC) of male rats. Reversing this effect with antidepressants, CMS increased 5-HT2Cr mRNA expression in the hippocampal CA4 region of both male and female rats. Overexpression of 5-HT1A in male mice was associated with a shorter immobility time in forced swimming test (FST) and contributed to the antidepressant response of citalopram (SSRI inhibitor) (Günther et al., 2011). Goodfellow et al. conducted an electrophysiological experiment on rat brain slices. The neurons of the prefrontal layer II/III vertebral body were directly inhibited by 5-HT1A, and the current of 5-HT1A was increased after ELS, and especially in females. This is the first electrophysiological examination of 5-HT1A (Goodfellow et al., 2009). Moreover, MAOA knockout mice are characterized by higher levels of serotonin and norepinephrine and increased aggressive behavior (Cases et al., 1995). For hyper-aggressive male mice who experienced peripubertal stress, MAOA expression and enzyme activity were reduced in the hypothalamus and were increased in the PFC. Hypomethylation in the PFC and hypermethylation in hypothalamus of the MAOA promoter were negatively associated with the expression pattern. In females, neither expression nor the epigenetic state of the MAOA gene was significantly altered between control and pre-pubertal stress (PPS) adult mice (Konar et al., 2019). In summary, the effects of ELS on 5-HT are diverse, and further analysis is needed. The 5-HTT± genotype was more sensitive to external stimuli. After ELS, 5-HT1A density was decreased, and 5-HT2Cr mRNA expression was increased, which may be helpful for systematic analysis of the human serotonin system.

## Sex Hormone

There have been a series of studies on the relationship between depression and sex hormones, and the studied hormones include estradiol, testosterone, rostenedione, estrone, 5β-dihydrotestosterone, etc. Lower estrogen levels in women and lower testosterone levels in men both could increase the risk of MDD (Bosch et al., 2006). The functions of sex hormones in neurotransmitters, neurotrophic factor expression, and neurogenesis are region and dose specific. The expression of 5-HTT was higher in the DRN (Savitz et al., 2009), and the activation of estrogen receptor leads to the increased release of 5-HT and its metabolite 5-HIAA in the DRN (Heikkinen et al., 2002) through a possible path in which ERβ enhances the activity of TPH directly (Wang et al., 2020). Besides, estrogen may also affect the serotonin receptor in the raphe nucleus, where E2 increases the combination of 5-HTT with SSRI antidepressant drugs (Krajnak et al., 2003). The deficiency of monoaminergic activity in the hippocampus is also related to depression, and estradiol has the same effect as SSRI on 5-HT1a. The mRNA expression of ERα in the amygdala of patients with MDD decreased significantly (Weiser et al., 2008), elevated by antidepressants. The expression of oxytocin (OXT) in hypothalamic paraventricular nucleus (PVN) of patients with depression was increased significantly, and androgen could directly inhibit the expression of the OXT gene by combining ARE with the human OXT gene promoter, leading to a potential neuroprotective effect. There are differences in sex, brain region, and depression subtypes between the activity of the OXT system and androgen in the pathogenesis of depression (Fernández-Guasti et al., 2000). There have been a variety of studies on the relationship between sex hormones and depression, but only a few on the role of sex hormones in depression caused by ELS. In addition, one of the manifestations of depression is a reduction in motivated behavior, which is controlled by the mesolimbic dopamine system, including projections from the ventral tegmental area (VTA) to the NAc and the PFC, with inputs from the medial preoptic area (mPOA). Estrogen and testosterone increase the release of dopamine (DA) in VTA and NAc, regulate the reuptake of DA, and increase the number of D1 and D2 receptors (Eck and Bangasser, 2020).

As for the treatment of MDD in humans, a large number of clinical trials have been conducted on the application of gonadal hormones, such as estrogen replacement therapy (ERT) or hormone replacement therapy (HRT = estrogen + progestin) for perimenopausal or postmenopausal, testosterone for depression symptoms in patients with HIV and AIDS, etc. (Dwyer et al., 2020). Treatment has not focused much on adolescent or adult depression caused by ELS.

### Studies on Animals

The effects of PNS on plasma sex hormones in male and female rodents were manifested as the decrease in the estradiol and testosterone in female rodents and the increase in testosterone in male ones. Postnatal early stress has no effect on estradiol levels in female rats, and the effect on testosterone in males is inconsistent. Sex hormones can produce effects on psychiatric disorders by enhancing the release of DA in the reward system and helping the sex differentiation in the brain (Lenz et al., 2011).

Cui et al. (2020) used maternal separation (MS) SD rats to simulate the ELS model, and they found that, although the MS group was more prone to depression and anxiety-like behavior, there was no significant difference in the level of sex hormones and their metabolites (including estradiol, testosterone, rostenedione, estrone, estriol, and 5βdihydrotestosterone) between the male MS group and the female MS group. Veenema found that MS could induce depression-like behavior and higher adrenocorticotropic hormone response to acute stressors. Arginine vasopressin (AVP) mRNA expression and AVP immunoreactivity were higher in the paraventricular and supraoptic nuclei of the hypothalamus of MS rats (Bosch et al., 2006). But the 5-HT immunoreactivity decreased in the anterior hypothalamus (Veenema et al., 2006). Reynaert employed rats in prenatal restraint stress (PRS) models and found that male PRS rats had increased levels of plasma dihydrotestosterone (DHT) and DA in NAc, and decreased levels of serotonin (5-HT) in NAc and PFC; female PRS rats had lower plasma estradiol levels (E2) and lower DA levels in NAc, as well as lower concentration of 5-HT in NAc and PFC. A supplement of E2 could reverse milk chocolate preference and the decrease of the 5-HT level in PFC. In the hypothalamus, PRS could increase the transcription levels of mRNA of ERα, ERβ, cocaine, and amphetamine receptor transcription peptide (CARTP) in males (Lund et al., 2006). At the same time, PRS could increase the mRNA level of the 5-HT2C receptor in females. All the changes were reversed by treatment of finasteride and E2, respectively (Reynaert et al., 2016). In female elderly rats, PRS could enhance the expression of MRs and brain-derived neurotrophic factor (BDNF) in the ventral hippocampus, besides enhancing the expression of glial fibrillary acidic protein (GFAP) and BDNF in the PFC (Verhaeghe et al., 2021). It was reported that there was still no significant difference in plasma sex hormone content between the MS group (the MS group: 14–16 days after birth, 6 h a day) and the control group, but ERβ played a key role through a DNA methylation mechanism in early stress-mediated emotion and emotion-induced late LTP in adult male rats hippocampus (Wang et al., 2013). In Kim et al.’s research, they studied the effect of ELS on the HPG axis and found that, after long-term stress or CORT treatment, the testosterone level in male Song Sparrows was increased, while GnRH supplementation did not increase the estradiol level in female Song Sparrows, but the stress and CORT treatment did. This study reveals that ELS could produce effects on HPG axis function (Schmidt et al., 2014). Exposure of lactating rats to social invasion could lead to inadequate care for F1 offspring and altered gene expression of OXT, prolactin (PRL), and AVP in female offspring (Carini and Nephew, 2013).

Failure of rodent ELS models to replicate the effects of sex factors may be associated with the insensitivity of rodents to behavioral measurement methods and the susceptibility of female rodents in different stages of the estrous cycle (Cui et al., 2020). These experiments suggested that stress could produce effects on sex hormones and sex hormone receptors in different brain regions (Reynaert et al., 2016).

**FIGURE 1:**
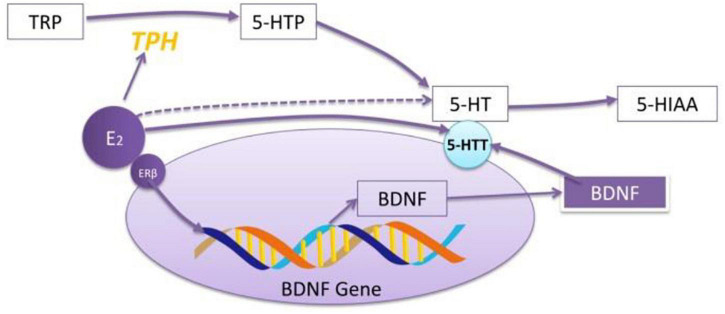
The role of E2 in regulating the effect of 5-HT. E2 increases the mRNA expression of TPH through exciting ERβ. ERβ regulates 5-HT through BDNF–5-HT2a pathway. E2 increases the reactivity of 5-HT to SSRI drugs. The effect of E2 on 5-HT depends on whether the model is established or not and the administration period.

Aggravation/remission of anxiety/depression after sex hormone injection: Nouri et al. (2020) treated NMRI mice with progesterone (10, 50, and 100 mg/kg, respectively, intraperitoneally) for 14 days, and they found that progesterone could significantly attenuates the depressive-like behaviors of MS and decrease the expression of inflammatory genes in the hippocampus. Estradiol can increase the mRNA expression of BDNF in the amygdala of ovariectomized rats, which is similar to the effect of antidepression (Borrow and Cameron, 2014) and upregulating the expression of 5-HTT through the path of BDNF (see Figure 1).

## Hypothalamic Pituitary Adrenocortical Axis Function

The HPA axis comprises the hypothalamic PVN, adenohypophysis, and adrenal cortex. PVN is the central part of the neuroendocrine axis. Its ascending fibers are connected widely with the amygdala complex, hippocampal formation, and marginal cortex of the limbic system. Its descending nerve fibers control the release of pituitary ACTH through CRH to regulate the synthesis and secretion of adrenal glucocorticoid (GC) (Lightman et al., 2020). HPA axis excitation can produce an obvious central effect during stress, resulting in emotional and behavioral changes like depression, anxiety, and anorexia. GC causes metabolic changes and anti-inflammatory effects, together with adverse reactions, including inhibition of immune response and gonadal axis, behavioral changes, such as depression and a suicidal trend (Arborelius et al., 1999; Van den Bergh et al., 2008; Cowen, 2010). MRs may be involved in regulating HPA axis function (Juruena et al., 2013). There is difference in HPA axis function between the neonatal period and the adult period for both humans and animals because the number and function of specific cells and receptors in the HPA axis for neonates are in the process of continuous development (van Bodegom et al., 2017), even though there are still sex differences in the HPA axis under physiological conditions and after stress in early life.

### Development of Hypothalamic Pituitary Adrenocortical Axis and Sex Differences

The fetal hypothalamus begins to form soon after the appearance of the hypothalamic sulcus in the 32-day embryo. The hypothalamus and pituitary portal vein systems develop mainly during the third trimester of pregnancy. Along with the ninth month of gestation, the maternal-placental – fetal steroid-producing unit activates the fetal HPA axis. CRH secretion by the placenta is related to the length of pregnancy. With the approach of delivery, CRH in maternal plasma increases and reaches its peak at delivery. Human placental CRH production may have evolved to stimulate fetal ACTH release and adrenal steroidogenesis, thus satisfying the high demand for synthesis of dehydroepiandrosterone (DHEA), the predominant source of placental estradiol (E2) (Gleason and Devaska, 2012).

### Sex Differences in the Amount and Function of CRH and CRHR

Hypothalamic pituitary adrenocortical axis excitation after stress can produce central effects, such as depression, anxiety, and anorexia. These effects are mainly caused by the increase of CRH secretion. During stress, the CRH neurons of PVA have a dense nerve fiber connection with the central nucleus of the amygdala complex. From the seventh month of pregnancy or earlier, the fetus can secrete CRH and ACTH (Fujioka et al., 1999) after maternal stress, raising fetal cortisol. After birth, CRH decreases to about 20% of adult levels, and the expression pattern of CRH is slightly different from adulthood, and CRH rises to the adult level 1 week after birth. CRHR is observed in gestational Day 17 (GD17) (Insel et al., 1988) and reaches more than 300% of adult levels during the first week after birth (Van Pett et al., 2000). At the same time, CRHR2 is temporarily expressed in mPFC but has disappeared in the later stage of SHRP (Gos et al., 2008). During the development of the amygdala, CRHR2 is expressed in different subregions. The central and basal parts begin to express CRHR2 on the 17th day of pregnancy and remain stable until adults. CRHR2 starts to express in the amygdala cortex after birth, increasing with age from the third day after delivery. CRHR1 mRNA in the hippocampus rises to the highest level at Day 6 after birth, reaching 300–600% of that in adults, and then decreased slowly; CRHR2 began to express from the first day after birth and remained stable during development (van Bodegom et al., 2017).

#### Studies on Humans

After controlling factors such as trauma type, severity, and post-traumatic support, women who experienced ELS were more likely to suffer from PTSD than men (Ordaz and Luna, 2012). Elevated plasma ACTH and salivary cortisol can be observed after PTSD (D’Elia et al., 2021). High estrogen levels elevated susceptibility to stress together with higher GC levels for unknown reasons (Dunlop and Wong, 2019). De Bellis et al. (1994) found that child sexual abuse was associated with mental illness after adulthood. The test was conducted on girls who had suffered sexual abuse, and ordinary girls were recruited as the control group. After administration of ovine CRH (oCRH), the amount of ACTH and total free cortisone in plasma, psychological status, and 24-h urinary free cortisone from the girls of both groups were measured. Compared to the control group, the girls who had suffered sexual abuse had higher proportion of suicide concepts, suicide attempts, and bad moods; and they also had lower levels of ACTH regardless of basal or induced by oCRH. However, there was no difference in plasma cortisone and 24-h urinary free cortisone regardless of basal and induced by oCRH between these two groups. The experiment verified that the adenohypophysis gland had a low response to oCRH. The possible mechanism was that ovarian hormones upregulated the CRH secretion, just as what was verified before (Dunlop and Wong, 2019). The function of the CRH and HPG axis is bidirectionally regulated. CRH inhibits GnRH in the hypothalamus through CRHR1 and CRHR2 in direct or indirect manners. Estrogen acts on the promoter region of the CRH gene in the hypothalamus and increases CRH transcription. Androgen inhibits CRH transcription by combining with the CRH promoter region (Ni and Nicholson, 2006). Previous studies have shown that HPA activity is enhanced in boys after stress before puberty, but it is supposed that sex hormones do not play a role at this stage. More studies have shown that HPA activity was enhanced after CRH stimulation in pre-pubertal males (Dahl et al., 1992) and post-pubertal females (Greenspan et al., 1993).

#### Studies on Animals

Previous studies have shown that early damage will affect the function of the HPA axis, such as the decrease of CRF and ACTH in animal models exposed to inflammatory pain and the decrease of the serum corticosterone level in male rats during recovery. Besides, ELS was related to the higher level of basic CRH, increased apoptosis of PVN (Tobe et al., 2005), increased expression of CRH and AVP mRNA, and increased expression of CRH2 mRNA in the medial BLA in females (DeSantis et al., 2011).

### Sex Differences in the Level and Function of ACTH

#### Studies on Humans

Patients with MDD with childhood trauma (CT) have a low baseline cortisol level, together with reduced ACTH levels at the baseline and Trier Social Stress Test (Mayer et al., 2020). PS causes a higher basic level of ACTH in animals (Lolait et al., 2007). Using nesting restriction and a maternal substitute, Fuentes et al. (2018) found that the concentrations of ACTH and cortisol in female serum before stress were higher than those in males and continued to increase after stress in both sexes. Slotten et al. (2006) used MS to establish a rat depression model and found that the basal GC and ACTH of males were lower than those of females.

### Differences in CORT and Glucocorticoid Receptor

Before birth, GRmRNA expression in hippocampus, mPFC, amygdala, PVN, and anterior pituitary is lower than those in adults (Pryce, 2008), so the negative feedback sensitivity of the HPA axis is lower. GR begins to rise in the second trimester of pregnancy and ends in PVN, pituitary, and hippocampus sequentially (Diaz et al., 1998). After birth, GR continues to develop. As for MRmRNA, its expression begins to rise in the second trimester of pregnancy and remains low until the third trimester of pregnancy. The content of MRmRNA in the hippocampus is almost the same as that in adults when GR is only 30% of that in adults, so the MR/GR ratio is very high. In the amygdala, the development of GR and MR is in the same pattern. During development, the level of GC increases significantly and the function of HPA matures. Studies on GR during puberty are still insufficient. It is reported that GR can reverse the effects of ELS during puberty (McCormick et al., 2010).

#### Studies on Humans

A study in Dutch showed that children with CT had a significantly higher level of cortisol arousal response and cumulative HPA axis markers than those without CT (Kuzminskaite et al., 2020). Nia Fogelman illustrated that there were clear sex differences in cortisol arousal response and the baseline, and no difference between non-stress state and response to stress (Fogelman and Canli, 2018). In the adrenal cortex, the 11β-hydroxysteroid dehydrogenase type 1 enzyme, which converts cortisone to cortisol, is encoded by HSD11B1, a variant of which is significantly associated with an increased risk of at least one suicide attempt and is possibly a relevant biomarker (Figaro-Drumond et al., 2020). Grunau reported that the increase in the number of injections in preterm infants was associated with a decrease in cortisol levels in preschool boys, but no difference was seen in basic cortisol levels in the control group (Mooney-Leber and Brummelte, 2020). Above all, stress during childhood results in CORT abnormality with sex differences.

#### Studies on Animals

During estrus, plasma and adrenal CORT were the highest in the ovarian cycle, suggesting the relationship between the ovarian hormone and HPA activity. Both PVN injection of estrogen and ERα agonists could increase the levels of GC, while injection of antagonistic agents was on the contrary. These results indicated that E2 could reduce the negative feedback effect of HPA through ERα (Zhang et al., 2009). At the same time, ERs and diarylpropionitrile (a non-steroidal estrogen receptor ER β selective ligand) directly increased CRF expression in hypothalamic 4B cells. Estradiol decreased the expression of GR and increased the activity of protein phosphatase 5, which inactivated GR (Chaloner and Greenwood-Van Meerveld, 2013). Brydges et al. (2020) used forced a swimming test, restraint, and other methods to create PPS models in rats, and they found that PPS could cause an increase of FKBP5 and AVPR1a in the female hippocampus and a decrease of GR and AVPR1a in mPFC. In the PFC of female rats, PPS triggered the increased expression of GR, the increased ratio of glucocorticoid to a mineralocorticoid (GR: MR) receptor, and decreased AVPR1a expression; in male rats, PPS induced the increased expression of GR, MR, FKBP5, and the oxytocin receptor in the hippocampus (Brydges et al., 2020). Pisu et al. (2016) used weaning to simulate the social isolation model of rats, and revealed that, after weaning, the male rats manifested a decreased number of isopregnanol ketone and plasma GC, and an increased level of plasma GC and BDNF after acute stress. Mooney-Leber et al. (2018) administered female and male rats with repeated needling with or without maternal care 4 days after birth (through a novel tea-ball infuser encapsulation model), and then they measured the plasma GC levels in adults with or without stress. The results showed that the basal glucocorticoid level of female adult rats was higher than that of male rats, and the GC level was significantly higher than that of non-stressed rats 1 h after stress. In addition, some studies have extended the scope of ELS to the fetal period, that is, to study the effects of stress on the fetus during pregnancy after adulthood of the examinee (Mooney-Leber and Brummelte, 2020). Brunton made the native lactating rats experience social frustration for more than 5 days through a pregnant intruder from its last week of pregnancy and studied the response of HPA to stress and anxiety-related behaviors in the adult offspring of social losers. The results showed that, in the progeny of PNS, the HPA response of female offspring to stress was significantly enhanced compared with that of males (Brunton and Russell, 2010). To sum up, the results of animal studies are inconsistent, since many studies have been conducted in this area and the results are stable; it is possible to employ animal experiments to explore the underlying mechanism. Women exposed to PPS did not show the usual increase in the plasma corticosterone level. Higher expression of oxytocin receptors was observed in the PFC, as well as higher AVPR1a and oxytocin in the hypothalamus, while men showed higher expression of GR, MR, GR: MR, FKBP5, and oxytocin receptors in the hypothalamus. These results indicated increased reaction of the female HPA axis to PPS and may further help explain why women show a higher susceptibility to certain stress-related psychopathologies in humans (Brydges et al., 2020).

## Genes and Epigenetics

Gene–environment interaction plays a vital role in mental diseases, and is also a research hotspot. ELS affects epigenetic modification, including DNA methylation, post-translational histone modification (methylation, phosphorylation, acetylation), and non-coding RNA. These modifications are stable, inducible, and reversible, which is why short-term environmental stimulation leads to lasting changes in gene expression and behavior (van Bodegom et al., 2017). In the common situation without stress, there are sex differences in the transcription of some genes that influence behavior. Some of these genes are sex hormone related, so transcription varies across menstrual cycles in women and across estrus cycles in animals. It is helpful to know that differentially expressed genes between the preestrus and interestrus are 20 times more than those of the females and males (Iqbal et al., 2020).

**FIGURE 2:**
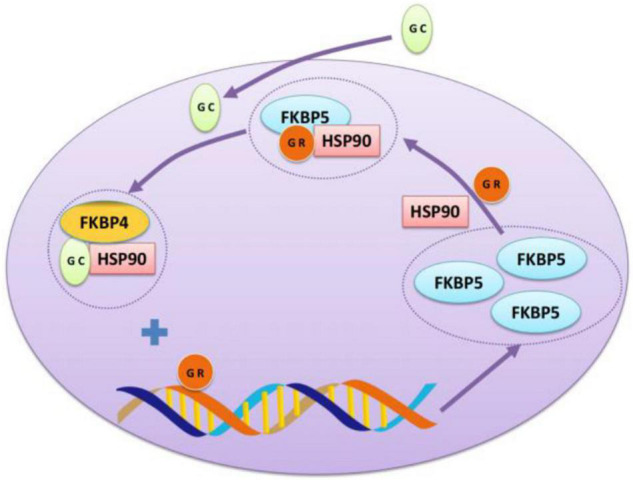
The interaction between GR and FKBP5. Glucocorticoid receptor (GR) can form a chaperone complex with FKBP5 and heat shock protein 90 as an intracellular receptor to prevent GR from being transported to the nucleus. After binding glucocorticoid (GC), GR dissociates and binds to the fkkbp5 enhancer-binding site, produces more FKBP5, conducting a negative feedback loop.

Previous studies have shown that the inhibition of negative feedback on the HPA axis can be completed by GR, which is a kind of an intracellular receptor regulated by a series of chaperone proteins (Rein, 2016). The most important part in the feedback is FK506 binding protein 51 (FKBP51) encoded by the FKBP5 gene. Fkbp51 plays a key role in regulating GR sensitivity as the chaperone protein of heat shock protein 90 (Hsp90) and GR. Once activated, GR enters the nucleus and binds to the glucocorticoid response factor in the enhancer region of FKBP5 to induce FKBP5 gene expression. Fkbp51 binds to GR and inhibits its function, which is the mechanism of GR desensitization (Rein, 2016; see Figure 2). Therefore, FKBP51 is the key factor in HPA axis adjustment (Wang et al., 2018). In the absence of glucocorticoids, FKBP5 is integrated into GR through Hsp90, which is composed of a mature GR heterologous complex (Wochnik et al., 2005). The complex remains inactive in the cytoplasm and has a low affinity for its ligands (Binder et al., 2008). Induced by glucocorticoids, FKBP5 is replaced by FKBP4, and steroid receptors are activated, allowing GR maturation and nuclear translocation as well as further genetic transcription (Xu et al., 2017).

First, FKBP5 gene variation and ELS may increase the risk of emotional disorders, resulting in a series of behavioral phenotypic abnormalities. Rs160780 of the FKBP5 gene has significant interaction with childhood adversity in brain white matter integrity involved in emotional processing (Kim et al., 2019). In a meta-analysis of the relationship among FKBP5 gene variation, ELS and depression/PTSD, it was found that those carrying T allele in the rs1360780 gene, C allele in the rs3800373 gene, or T allele in the rs9470080 gene had a higher risk of depression or PTSD after early trauma (Dackis et al., 2012). There are also studies showing that patients with MDD have smaller hippocampal volume, especially in the corn ammonis and dentate gyrus. Moreover, it was also found that those with the T allele have smaller volumes in the hippocampal amygdala transition region (HATA) than those homozygous with the C allele (Mikolas et al., 2019). In addition, it was revealed that FKBP5 gene variation, combined with subjective or objective ELS, could predict more obvious depressive symptoms in their middle age (Lahti et al., 2016). In research in which DNAm in peripheral blood samples after exposure to dexamethasone was measured using human peripheral blood samples, 1, 3, and 6 h after dexamethasone treatment, differentially methylated CPGs were detected in enhancers co-located with GC receptor-binding sites and returned to a normal level within 23 h (Wiechmann et al., 2019). Using the pixel types estimated from Illumina 450k array data, it was observed that age (seven sites), sex (five sites), smoking (six sites), BMI (eight sites), major depression disorder (MDD) (four sites) all could produce significant effects on methylation (Wiechmann et al., 2019). In addition to GR, FKBP5 also interacted with other molecular chaperones, including MR, progesterone receptor, estrogen receptor and androgen receptor. Similar to its effect on GR, FKBP5 inhibited MR and PR activities and promoted ER and AR activities (Zannas et al., 2016). Fkbp51 seems to positively regulate the activity of androgen receptors (Ni et al., 2010). Both natural and synthetic androgens can upregulate the expression of FKBP51 through AR binding to the enhancer region of the FKBP5 gene (Davies et al., 2005), suggesting that this may be an automatic regulatory pathway aimed at enhancing androgen sensitivity, which makes FKBP51 a potentially important factor in the etiology of prostate cancer (Maeda et al., 2022). Fkbp51 plays a role in enhancing AR function, indicating that FKBP51 can directly or indirectly target the ligand-binding domain of GR and AR, but the exact molecular mechanism remains unclear (Stechschulte and Sanchez, 2011). In addition, FKBP51 may be an intermediate factor in the regulation of ER activity. It is reported that tetrapeptide repeat domain 9A (TTC9A) may negatively regulate the activity of ERα by interacting with chaperones such as FKBP38 and FKBP51 (Shrestha et al., 2015). In conclusion, FKBP5 is involved in sex differences of ELS-induced depression through regulating sex hormone receptor activity.

DNA methylation is a kind of epigenetic modification, in which methyl is added to the cytosine of DNA sequence. It is usually related to the inhibition of gene expression, but, in a few cases, the expression would be increased (Bird, 2002). It can occur on C-G dinucleotide and, occasionally, on other dinucleotides. Methyl was added by DNA methyltransferases DNMT1, DNMT3a, or DNMT3b. DNA demethylation is carried out by oxidation of methyl or by enzymes such as Gadd45b (Mo et al., 2015). Methylation is accomplished by MeCP2, which binds to methylated DNA, assists gene silencing, or assists gene expression through the recruitment of corepressors or coactivators (Doherty and Roth, 2018). The response to stress is also related to CRF promoter methylation. CRF expression in the hypothalamus of stress-sensitive mice was higher than that of stress-tolerant mice due to decreased methylation of CRF promoters (Mueller and Bale, 2008). Antidepressant therapy that blocks the methylation of CRF promoters would improve the loss of social interest. Injecting siRNA sequence into hypothalamus would enhance the tolerance of CRF promoter. The individual differences of CRF signals are regulated by the epigenetic regulation of CRF promoter, and there are sex differences (Hodes and Epperson, 2019).

There are few studies exploring the relationship between DNA methylation and non-coding RNA related to histone modification and depression in humans, so the relationship between histone modification and non-coding RNA and depression is still unclear. Many animal studies have shown that mRNA expression of histone deacetylases (HDACs) 1, 3, 7, and 8 decreased, while the expression of acetylated histone H4 protein increased in the prefrontal lobe of MS mice. The increase of HDACs expression was observed in the VTA of MS rats (Li et al., 2020), while the sex difference in arginine methylase (H3K) was observed in different subtypes, experimental animals, modeling methods, as well as different brain regions (Torres-Berrío et al., 2019; Kronman et al., 2021).

It is indisputable that epigenomes have sex duality in many mechanisms in the development and the whole life cycle. It has been reported that there are sex differences in the expression of MeCP2, Gadd45b, and Dnmt3a in the amygdala of developing rats in the expression of MeCP2 in the ventral center of the hypothalamus and preoptic chiasma of developing rats, and in the methylation of steroid receptors in the brain region of sex duality. It is reported that different kinds of maternal care could lead to the difference in methylation patterns in the offsprings of adult male rats (Kurian et al., 2007; Kolodkin and Auger, 2011; Kigar et al., 2016; Doherty and Roth, 2018). It is reported that dogs experienced adverse living conditions outside the cage 7 days after birth, and sex-specific changes could be seen in mPFC apparent regulators 90 days after birth (Burns et al., 2018). Additionally, there are sex differences in BDNF methylation, and the pattern displayed by females is very inconsistent with that of males. Grégoire et al. (2020) exposed CD1 mice to PNS and found that the level of total exon IV BDNF in the Hippo decreased.

MicroRNAs also play a subtle role in enhancing gene expression by reducing rather than eliminating specifically targeted mRNA transcripts, which may also be involved in the development of mental disorder through gene dysregulation involved in specific disease-related processes (Penner-Goeke and Binder, 2019). PS can change the characteristics of miRNA in specific parts of the brain, further affect offspring brain development and axon guidance, and lead to neurological diseases (Zucchi et al., 2013). The increased methylation of Hsd11b2 gene DNA in the placenta results from repeated stress on the mother. In the fetal hypothalamus, the methylation of the enhancer of the gene decreased, but the methylation of exon 1 increased but did not affect the expression of mRNA (van Bodegom et al., 2017). In male animals, sucrose preference was positively correlated with PFC miR-411-5p and negatively correlated with amygdala miR-133B-3p. In female animals, sucrose preference was negatively correlated with PFC miR-142-5p and miR-483-3p, miR-31A-3p, miR-466b-3p, and miR-483-5p in amygdala. A total of 88 miRNAs were significantly correlated with the escape time of male animals, most of which were in PFC. No miRNA expression was significantly associated with FST escape time in female animals (Yuan et al., 2018).

Except for epigenetics, the genome itself may directly lead to sex differences in neurotransmitters and behavior after stress (Howard et al., 2019). After early chronic stress, the sex chromosome group affects the expression of GABA, 5-HT, and dopamine-related genes in the prefrontal lobe. Compared with XX mice, XY mice had lower expression of the above genes, and the anxiety degree of XY mice without testosterone is higher than XX mice, suggesting that testosterone has an antidepressant effect, and the effect of gonadal hormone offsets the above gene expression changes caused by the sex chromosome group (Seney et al., 2013).

In addition, BDNF, NPY, TNF, and inflammatory factors have been reported to be involved in ELS-mediated depression regulation (Park and Bowers, 2010; Quinn et al., 2012; Alviña et al., 2021). The expression of BDNF is related to the pathogenesis of depression. For MS mice, the expression of BDNF in the hippocampus and striatum increased significantly in males, while the expression of BDNF in the striatum in stressed female mice decreased significantly, and there was no difference in the expression of BDNF in the frontal cortex and hippocampus (Luoni et al., 2016). Estrogen may also indirectly affect serotonin activity by changing the expression of BDNF. BDNF knockout mice show a decrease in 5-HT1A binding and mRNA expression, while central BDNF injection increased 5-HT1A hippocampal receptor gene expression (Borrow and Cameron, 2014). González-Pardo et al. (2020) applied the MS animal model to evaluate regional brain mitochondrial function, monoaminergic activity, and neuroinflammation, and they found that TNFα and IL-6 levels in the PFC and hippocampus of MS male rats were increased (Rothwell and Luheshi, 2000; Heida and Pittman, 2005). These results reveal that ELS could produce complex long-term effects on sex and brain regions (González-Pardo et al., 2020). Babri et al. (2014) explored whether the exposure of neonatal mice to TNFα would affect body weight, stress-induced corticosterone (COR), anxiety, and depression-like behaviors in adult mice. As a result, they found that neonatal TNFα treatment could reduce the weight of male and female newborns. A high dose of TNF can increase the stress-induced COR level, anxiety, and depression-like behaviors of adult male mice. Therefore, it is inferred that exposure to TNFα during the neonatal period can alter brain and behavioral development in a dose- and sex-dependent manner (Babri et al., 2014).

## Conclusion and Expectation

Early life stress can cause depression in childhood or pre-puberty, and the depression is sex specific. The mechanisms underlying the difference between different sexes are that there is sex duality in the serotonin system in 5-HT synthesis rates, 5-HT metabolite levels, receptor- and transporter-binding potentials, SSRI responses, and tolerance. The methylation levels of the 5-HTT gene are different between men and women. Moreover, E2 might be helpful to increase the level of 5-HT and the expression of the 5-HT receptor. Besides, there are sex differences in CRH expression caused by severe trauma, in HPA axis function after stress, i.e., a trend of plasma CORT and its receptor. In addition, FKBP5 can enhance the activities of ER and AR. Furthermore, FKBP51 can negatively regulate ERα through chaperone protein interaction. There are sex differences in the expression of a variety of proteins or enzymes that affect epigenetics. BDNF, NPY, and TNF have been reported to be involved in the regulation of depression mediated by ELS, showing sex differences, too. The paper highlights the important role of ELS in human mental health and helps to screen possible clinically effective anti-depression targets, which will help promote the healthy development of children and adolescents.

## Author Contributions

XA and WG wrote the first draft. HW, XF, and ML provided writing reviewing and editing. YZ, YL, and RC provided conceptualization of ideas. WY, ZZ, and GZ provided supervision. All authors approved the final version of the manuscript for submission.

## Conflict of Interest

The authors declare that the research was conducted in the absence of any commercial or financial relationships that could be construed as a potential conflict of interest.

## Publisher’s Note

All claims expressed in this article are solely those of the authors and do not necessarily represent those of their affiliated organizations, or those of the publisher, the editors and the reviewers. Any product that may be evaluated in this article, or claim that may be made by its manufacturer, is not guaranteed or endorsed by the publisher.
